# Maternal High-Fructose Intake Activates Myogenic Program in Fetal Brown Fat and Predisposes Offspring to Diet-Induced Metabolic Dysfunctions in Adulthood

**DOI:** 10.3389/fnut.2022.848983

**Published:** 2022-04-11

**Authors:** Peng Wang, Tian Wu, Qinghua Fu, Qichao Liao, Yan Li, Tengda Huang, Yixing Li, Lei Zhou, Ziyi Song

**Affiliations:** State Key Laboratory for Conservation and Utilization of Subtropical Agro-Bioresources, College of Animal Science and Technology, Guangxi University, Nanning, China

**Keywords:** maternal high-fructose intake, offspring, brown fat, myogenic pathway, metabolic dysfunctions

## Abstract

Excess dietary fructose intake is a major public health concern due to its deleterious effect to cause various metabolic and cardiovascular diseases. However, little is known about the effects of high-fructose consumption during pregnancy on offspring metabolic health in adulthood. Here, we show that maternal consumption of 20% (w/v) fructose water during pregnancy does not alter the metabolic balance of offspring with a chow diet, but predisposes them to obesity, fatty liver, and insulin resistance when challenged by a high-fat diet. Mechanistically, diet-induced brown fat reprogramming and global energy expenditure in offspring of fructose-fed dams are impaired. RNA-seq analysis of the fetal brown fat tissue reveals that the myogenic pathway is predominantly upregulated in the fructose-treated group. Meanwhile, circulating fructose level is found to be significantly elevated in both fructose-fed dams and their fetuses. Importantly fructose gavage also acutely activates the myogenic program in mice brown fat. Together, our data suggest that maternal high-fructose intake impairs fetal brown fat development, resultantly attenuates diet-induced thermogenesis and causes metabolic disorders in adult offspring probably through inducing myogenic signature in brown fat at the fetal stage.

## Introduction

The global epidemic of obesity and type 2 diabetes mellitus (T2DM) remains a major public health issue. Numerous studies have demonstrated that the nutritional environment that an individual is exposed to before birth is a critical determinant of their risk of developing obesity and metabolic diseases in their later life (1, 2). Exposure to unbalanced maternal nutrition, particularly overnutrition, is associated with altered development of key physiological systems, which predisposes them to adult onset of non-communicable diseases, such as obesity, T2DM, fatty liver, and cardiometabolic disease (1, 3–5).

Among the specific nutritional factors, excess fructose consumption is increasingly considered as a major contributor to the emerging epidemics of obesity and the associated metabolic diseases (6–9). Fructose is a simple sugar found naturally in honey, fruit, and some vegetables. Over the last decade, fructose consumption has increased significantly and has become the most typical sugar consumed by man, owing to its use as a sweetener in processed foods and soft drinks in the form of sucrose or high-fructose corn syrup (9). The increasing prevalence of soft-drinks consumption results in a higher incidence of pregnant women having excess fructose intake. However, so far the consequence and mechanism of maternal high-fructose intake on offspring metabolic health in later life are still unclear, albeit existing of few animal studies (10–14).

In adult obesity or obesity “programmed” by early-life insults, adipose tissue dysfunction is considered to be an important contributor to the metabolic alterations in humans and rodents (15). There are mainly two types of adipose tissues, namely, white adipose tissue (WAT) and brown adipose tissue (BAT) (16), which have distinct characteristics and functions. WAT is responsible for energy storage in the form of triglyceride (TG), while BAT dissipates energy in the form of heat because of the presence of abundant mitochondria and uncoupling protein 1 (UCP1) (16). Brown adipocytes derive from a common pool of precursors with skeletal muscle during early development, with the divergence occurring between embryonic day (E) 9.5 and E12.5 in mice (17). Disturbance of the divergence by genetic or nutritional manipulations leads to abnormal BAT development and results in offspring obesity and metabolic disorders in later life (18–21). However, up to date, there is a paucity of data examining the impacts of increased fructose intake during pregnancy and subsequent effects on fetal BAT development and offspring energy metabolism later on.

Therefore, this study was designed to reveal the impacts and potential mechanism of maternal high-fructose intake on offspring metabolic health, with an emphasis on fetal BAT development and its associated energy metabolism in adulthood. We have found that maternal high-fructose intake during pregnancy promotes a myogenic program in fetal BAT, resultantly impairs high-fat diet (HFD)-induced BAT thermogenesis and exacerbates HFD-induced obesity and metabolic disorders in adult offspring.

## Materials and Methods

### Animal Studies

All animal studies were according to protocols approved by the Animal Ethics Committee of Guangxi University (GXU2021-123). Twelve-week-old C57BL/6J mice were purchased from the Guangxi Medical University (Nanning, China) and housed under controlled light and temperature conditions (12-h light/dark cycle; 22 ± 2°C). After 1 week’s adaption, the mice were mated for one night. Success in mating was confirmed by the presence of a vaginal plug, and then pregnant mice were randomly assigned to either sterilized tap water or 20% (w/v) fructose (Sigma, F3510) water until delivery. At E18.5, a subset of pregnant mice was killed and the BAT of male fetuses was collected for further analysis. Fetal sex was identified by PCR (22). The remaining female mice were allowed to give birth. On the day of birth, litter sizes were balanced to five pups. All pups were weaned on postnatal day 21 and separated into two groups at 7 weeks old, one group was fed a regulated chow (10% kcal energy from fat) and the other group was fed a HFD (60% kcal energy from fat). To avoid confounding sexual effects, only male offspring were used in this study.

### Glucose and Insulin Tolerance Test

A glucose tolerance test (GTT) was performed on 15-week-old mice after a fast for 15 h followed by an intraperitoneal injection of glucose (1.5 g/kg D-glucose). One week later, an insulin tolerance test (ITT) was performed after a fast for 5 h followed by an intraperitoneal injection of insulin (1 U/kg insulin). The blood glucose level was measured by tail bleeding at 0, 15, 30, 60, 90, and 120 min after glucose or insulin injection using a glucose meter.

### Body Composition and Indirect Calorimetry Analysis

The fat mass and lean mass of the mice were analyzed once a month through NMR (Niumag QMR23-060H-I, Suzhou, China) following the instruction of the manufacturer. At 16–17 weeks of age, indirect calorimetry measurements were performed using the Promethion Metabolic Cage System (Sable Systems International, Las Vegas, NV, United States). Mice were acclimatized for 24 h in the Promethion system before the measurement was started. Instrument control and data acquisition were performed according to the instructions of the manufacturer. Raw data were processed using ExeData software (Sable Systems).

### Measurement of Plasma and Hepatic Parameters

Triglyceride (TG) and total cholesterol (TC) content in plasma and liver tissues were measured by TG assay kit (A110-1-1, Nanjing Jiancheng Bioengineering Institute, China) and TC assay kit (A111-1-1, Nanjing Jiancheng Bioengineering Institute, China) according to the instructions of the manufacturer. For tissue samples, protein concentrations were measured by using the BCA protein quantitative assay kit (Beyotime Biotechnology, Shanghai, China), and the TG and TC data were expressed as μmol/g protein. Fructose level in plasma was determined by fructose assay kit (G0530W, Shanghai Jianglai Biotechnology Co., Ltd., Shanghai, China) by following the instruction of the manual and the optical density was evaluated at 450 nm by using the continuous spectrum microplate plate reader (Epoch) (Bio Tek Instruments, Inc.).

### H&E Staining and Oil Red O Staining of Mouse Samples

Livers and adipose tissues were dissected and fixed in tissue-fixing liquid overnight at 4°C for the paraffin section and frozen section. The paraffin sections were stained with H&E and the frozen sections were stained with Oil Red O. Sample sectioning and staining were performed at the Wuhan Servicebio Technology Co., Ltd. (Wuhan, Hubei, China). Images were collected by light microscope (Biological microscope ML31, MSHOT, Guangzhou, China) and cell area was analyzed by ImageJ software.

### RNA Extraction and Quantitative Real-Time PCR

RNA extraction from BAT tissues was performed by re-suspending 10–20 mg of frozen tissues in 1 ml Trizol (Life Technologies, United States), and lysed by using a tissue lyser (Qiagen, Germany) for 3 min at 30 Hz. Then, total RNA was isolated following Trizol manufacturer’s instructions. The purity and concentration of the total RNA were determined by Tecan infinite M200 Pro (Grödig, Austria). cDNA was synthesized from 1 μg of total RNA using RevertAid First Strand cDNA Synthesis Kit (Thermo Fisher, United States). Real-time qPCR was performed using a 2× RealStar Green Fast Mixture (GenStar, Beijing, China). All data were normalized to the *Tbp* level and analyzed following the 2^–ΔΔ*Ct*^ method. The sequences of primers used are listed in Supplementary Table 1.

### RNA-Seq Analysis

Purified total RNA samples from E18.5 fetal BAT were sent to the Guangzhou Gene *Denovo* Biotech Co. Ltd. (Guangzhou, China) for library construction and sequencing by using the Illumina novoseq 6000 platform. The raw RNA-seq data were submitted to the Gene Expression Omnibus data repository^1^ with the login number GSE193031. The procedure of data analysis is as follows: first, the high-quality sequences, which were filtered by Fastp (v0.23.2), were mapped to the mouse reference genome (mm10) by Bowtie2 (v2.0.0.5) and Tophat (v2.0.0). Then, the mapping readings were assembled and GTF files were generated by StringTie (v2.2.0). The GTF files were then merged into one file by Cuffmerge, from which the counts of each gene were extracted. Differentially expressed genes were analyzed based on the gene counts by using a RNA-seq processing tool DESeq2 and selected by *p*-value < 0.05 and fold change (FC) >1.3, and visualized using R tools ggplot2. For Gene Set Enrichment Analysis (GSEA), the normalized gene expression data were ranked according to the log2 FC and were visualized by ClusterProfiler (R package, version 3.18.1).

### Statistical Analysis

The results are expressed as mean ± SD. Significance was estimated by unpaired Student’s *t*-test (for two groups) or one-way ANOVA (for multiple groups). A probability of *p* < 0.05 was considered to be statistically significant. The statistical analysis and figures were prepared using GraphPad Prism 8.0.

## Results

### High-Fructose Intake During Pregnancy Does Not Alter Body Weight but Causes Slight Lipid Metabolic Disorders in Female Mice

**FIGURE 1:**
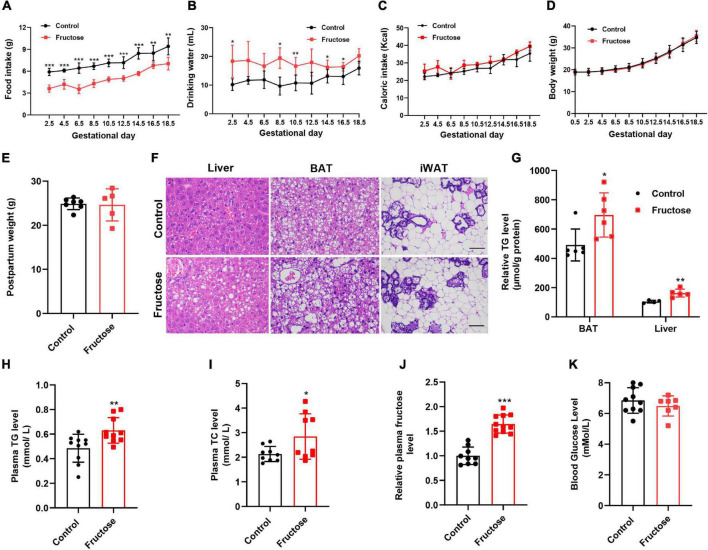
High-fructose intake during pregnancy does not alter body weight but causes slight lipid metabolic disorders in female mice. **(A–C)** Daily food intake, drinking water consumption, and total caloric intake of the control pregnant mice and the high-fructose-fed pregnant mice during the period of gestation (*n* = 5–6). **(D)** Bodyweight of the control pregnant mice and the high-fructose-fed pregnant mice during the period of gestation (*n* = 7–10). **(E)** Postpartum weight of the control pregnant mice and the high-fructose-fed pregnant mice (*n* = 5–7). **(F–K)** All data were from control pregnant mice and the high-fructose-fed pregnant mice at day 18.5 of pregnancy. Mice fasted for 4–6 h before sacrifice. **(F)** H&E staining of paraffin sections of liver, brown fat tissue (BAT), and inguinal white fat tissue (iWAT). Scale bar, 50 μm for liver and BAT, 100 μm for iWAT. **(G)** Quantification of triglyceride content in liver and BAT (*n* = 4–6). **(H,I)** Plasma concentrations of triglyceride (TG) and total cholesterol (TC) (*n* = 9–10). **(J)** Relative fasting plasma fructose level (*n* = 11). **(K)** Fasting blood glucose level (*n* = 7–10). *p < 0.05, **p < 0.01, ***p < 0.001.

In this study, C57/BL6J mice were used as an animal model to evaluate the effects of maternal high-fructose consumption in gestation on offspring metabolic health. First, we observed that 20% (w/v) fructose water administration significantly increased the water consumption, but remarkably decreased the food intake, by dams during the course of gestation (Figures 1A,B). However, intriguingly, total energy consumption analysis showed that the two groups of animals consumed equal amounts of energy (Figure 1C). Consistent with the energy intake, no difference was seen in the bodyweight and even in the postpartum weight between the two groups (Figures 1D,E). Impressively, the dams with high-fructose feeding showed slight fatty liver and lipid droplet-enlarged BAT, while leaving WAT less affected at 18.5 days of gestation (Figures 1F,G). Meanwhile, the mice also exhibited higher plasma TG and TC levels (Figures 1H,I). These data indicate that short-term high-fructose feeding has already disturbed maternal metabolic homeostasis. Next, given fructose is the leading factor to the metabolic phenotypes, thus we measured the circulating fructose level in dams. The result showed that plasma fructose level in fructose-fed dams was about 1.6-folds higher than that in the control mice (Figure 1J). By contrast, the blood glucose level was not altered by maternal fructose feeding (Figure 1K). Collectively, these data suggest that maternal high-fructose consumption causes gestational fatty liver and dyslipidemia but not obesity.

### Maternal High-Fructose Intake During Pregnancy Predisposes Adult Offspring to High-Fat Diet-Induced Obesity, Fatty Liver, and Insulin Resistance

**FIGURE 2:**
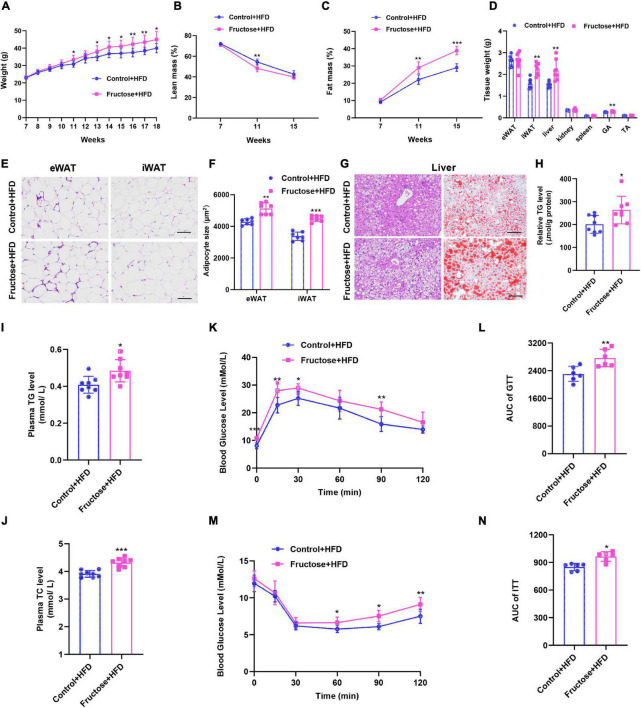
Maternal high-fructose intake during pregnancy predisposes adult offspring to HFD-induced obesity, fatty liver, and insulin resistance. All data were from the HFD-fed control offspring and HF offspring. **(A)** Bodyweight curve of the offspring. **(B,C)** Relative body composition of the offspring. **(D)** Tissue weights of the offspring. **(E)** H&E staining of paraffin sections of inguinal white fat (iWAT) and epididymal white fat (eWAT). Scale bar, 100 μm. **(F)** Quantification of average adipocyte area from images depicted in **(E)**. **(G)** H&E staining of paraffin sections of liver and Oil Red O staining of frozen section of the liver. Scale bar, 100 μm. **(H)** Quantification of triglyceride content in liver (*n* = 8). **(I,J)** Plasma concentrations of triglyceride (TG) and total cholesterol (TC) (*n* = 8). **(K)** Blood glucose concentrations during glucose tolerance test (*n* = 6). **(L)** Quantification of the area under the curve of **(K)** (*n* = 6). **(M)** Blood glucose concentrations during insulin tolerance test (*n* = 6). **(N)** Quantification of the area under the curve of **(M)** (*n* = 6). *p < 0.05, **p < 0.01, ***p < 0.001.

Next, to evaluate the effects of maternal high-fructose consumption on offspring metabolic health, offspring were placed on a chow diet (CD) or a HFD. On regular chow, offspring from dams with high-fructose intake (henceforth referred to as HF offspring) did not display any notable difference in metabolic phenotypes compared with the controls except the glucose tolerance (Supplementary Figure 1), indicating maternal high-fructose consumption has limited effects on offspring metabolic health under CD condition. However, when under HFD exposure, the HF offspring exhibited obvious metabolic disorders compared with the controls. First, the HF offspring gained more body weight, which was mainly due to increased fat mass but not lean mass (Figures 2A–C), indicating the HF offspring were prone to develop obesity than the controls on a HFD. Specifically, the weight of inguinal adipose tissue (iWAT) and liver was significantly higher in HF offspring, while interestingly, the weight of epididymal adipose tissue (eWAT) was similar between the two groups (Figure 2D). In agreement with the tissue weight, histological analysis showed that the cell size of iWAT, but not eWAT, was larger in HF offspring than in the controls (Figures 2E,F). Besides, the liver of HF offspring had accumulated more lipid droplets than the controls, which was further evidenced by Oil Red O staining and TG measurement (Figures 2G,H), suggesting the HF offspring developed more serious fatty liver. Furthermore, the HF offspring had high levels of plasma TG and TC (Figures 2I,J). In addition, we evaluated the glucose tolerance and insulin sensitivity of the offspring. As expected, the HF offspring showed significantly higher blood glucose concentrations than controls (Figures 2K,L). Similarly, the HF offspring exhibited impaired responses to insulin during an ITT (Figures 2M,N). Taken together, these data strongly suggest that maternal high-fructose consumption during pregnancy predisposes adult offspring to HFD-induced obesity, fatty liver, and insulin resistance.

### High-Fat Diet-Induced Energy Expenditure Is Impaired in Adult Offspring of Dams Fed With High Fructose

**FIGURE 3:**
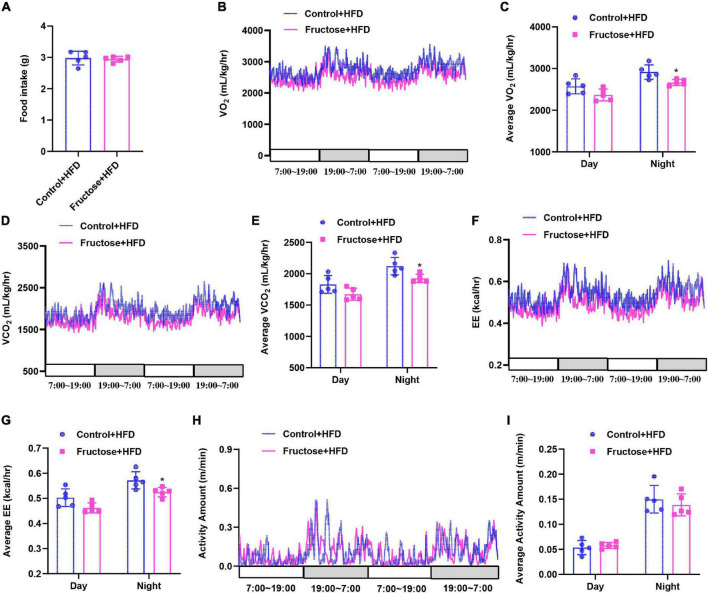
High-fat diet-induced energy expenditure is impaired in adult offspring of dams fed with high fructose. All data were from the 17-week-old HFD-fed control offspring and HF offspring. **(A)** Daily food intake of the mice (*n* = 5). Indirect calorimetry analysis of day and night oxygen consumption (VO_2_, **B,C**), carbon dioxide production (VCO_2_, **D,E**), heat production **(F,G)**, and physical activity **(H,I)** of the mice (*n* = 5). *p < 0.05.

Next, to investigate the mechanisms of HF offspring predisposing to HFD-induced metabolic disorders than the controls, we performed an indirect calorimetry assay as disturbed energy metabolism plays a key role in the development of metabolic dysfunction (19, 23). First, we found when on a CD there was no difference between HF offspring and controls in terms of energy intake, energy expenditure, or physical activities (Supplementary Figure 2). However, when under HFD challenge, HF offspring exhibited less oxygen consumption, carbon dioxide release, and heat production compared with the controls, with the exception of food intake and physical activities (Figure 3). Thus, these findings suggest that maternal high-fructose consumption results in impairment of diet-induced energy expenditure in HF offspring.

### High-Fat Diet-Induced Thermogenic Program of Brown Fat Is Attenuated in Adult Offspring of Dams Fed With High Fructose

**FIGURE 4:**
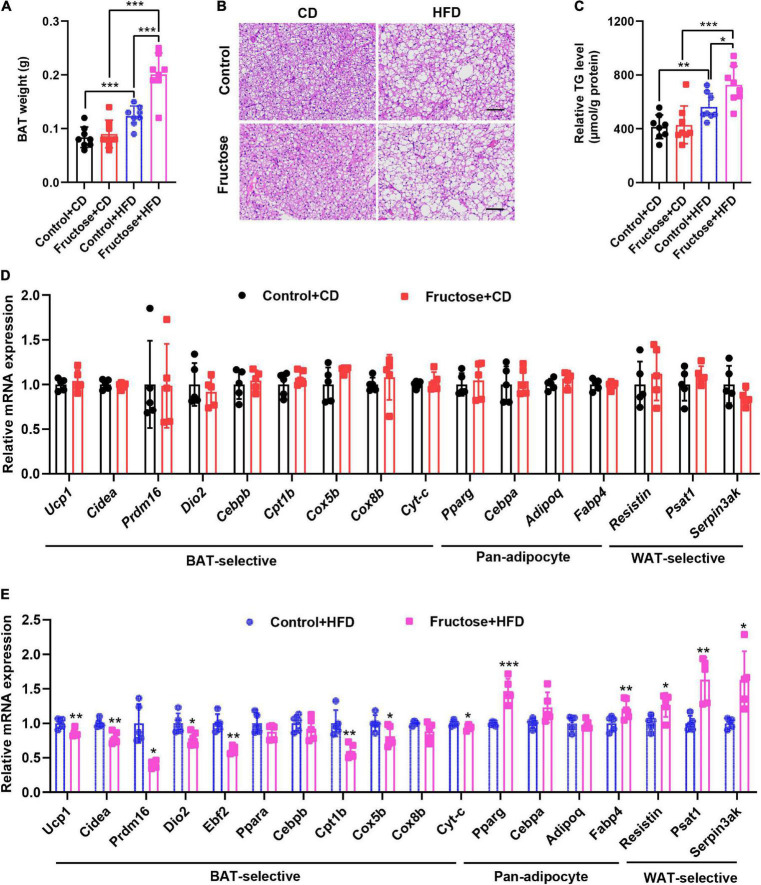
High-fat diet-induced thermogenic program of brown fat is attenuated in adult offspring of dams fed with high fructose. **(A)** The weight of BAT tissue in CD-fed or HFD-fed control offspring or HF offspring. **(B)** H&E staining of paraffin sections of BAT. Scale bar, 100 μm. **(C)** Quantification of triglyceride content in BAT (*n* = 8). **(D)** qRT-PCR analysis of BAT-selective genes, pan-adipocyte genes, and WAT-selective genes in BAT from CD-fed control offspring or HF offspring (*n* = 5). **(E)** qRT-PCR analysis of BAT-selective genes, pan-adipocyte genes, and WAT-selective genes in BAT from HFD-fed control offspring or HF offspring (*n* = 5). *p < 0.05, **p < 0.01, ***p < 0.001.

As brown fat is highly involved in the regulation of thermogenesis, glucose metabolism, and insulin sensitivity (24, 25), we then investigated whether the BAT function of offspring was adversely affected by maternal high-fructose feeding. First, we examined the mass of the BAT depot, and found the BAT weight of HF offspring was similar to the controls under CD condition; however, it significantly increased when under HFD feeding (Figure 4A). Moreover, we found that this increase in BAT weight was mainly due to the increased lipid content as showed by H&E staining and TG-level measurement (Figures 4B,C). Since the higher TG content in BAT is usually associated with its lower thermogenic activities (26), we then analyzed the expression of a panel of BAT marker genes in offspring BAT. On the CD, we did not notice any difference in mRNA levels of the genes, namely, BAT-selective genes, pan-adipocyte genes, and WAT-selective genes (Figure 4D). However, when under HFD condition, the mRNA levels of BAT-selective genes were significantly lower in HF offspring than the controls, whereas the mRNA levels of common adipogenic genes and WAT-specific genes were significantly upregulated in BAT of HF offspring compared with the controls (Figure 4E). Collectively, these data indicate that maternal high-fructose feeding blunts HFD-induced BAT reprogramming and thermogenic function in offspring, which could be responsible for the suppression of energy expenditure in HF offspring.

### Maternal High-Fructose Intake During Pregnancy Induces Myogenic Signature in Fetal Brown Fat

**FIGURE 5:**
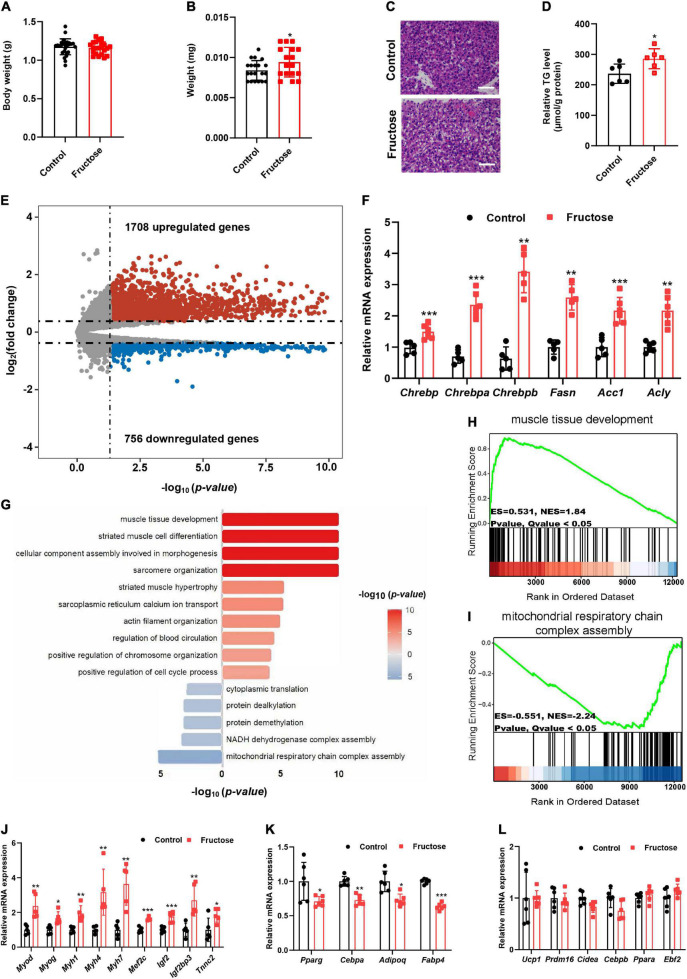
Maternal high-fructose intake during pregnancy induces a myogenic signature in fetal brown fat. All data were collected from fetuses at E18.5. **(A)** Bodyweight of fetuses (*n* = 22–27). **(B)** Fetal BAT weight (*n* = 17–20). **(C)** H&E staining of paraffin sections of fetal BAT. Scale bar, 50 μm. **(D)** Quantification of triglyceride content in fetal BAT (*n* = 6). **(E)** Volcano plot comparison of genes in fetal BAT regulated by maternal high-fructose versus the control (*n* = 2 per group). **(F)** qRT-PCR analysis the levels of *Chrebp* and its target genes in fetal BAT (*n* = 5–6). **(G)** Gene Set Enrichment Analysis (GSEA) of differentially expressed genes. **(H,I)** GSEA analysis of the most significant upregulated and downregulated pathways. **(J–L)** qRT-PCR analysis of myogenic genes, pan-adipocyte genes, and brown fat selective genes in fetal BAT (*n* = 5–6). *p < 0.05, **p < 0.01, ***p < 0.001.

Next, to investigate how maternal high-fructose feeding causes adult offspring BAT dysfunction, we switched our focus on the fetal stage since the fructose was only administrated to the dams in the gestation period. Thus, fetuses and their BAT at E18.5 days were isolated and analyzed. No difference was observed in total fetus weight (Figure 5A), but a significant increase was seen in the fetal BAT weight at the end of gestation (Figure 5B). Microscopic examination detected the emergence of a lot of adipocytes containing single lipid droplets only in fetal BAT from fructose-fed dams but not from the control dams (Figure 5C). Consistently, TG level was also significantly increased in fetal BAT of fructose-fed dams (Figure 5D). These data suggest that the fetal BAT from fructose-fed dams has lipid metabolic disorder. Next, to identify genes or pathways that contribute to this phenotype, we, therefore, analyzed the global gene expression profiles of the fetal BAT, and found a total of 2,464 genes that were differentially expressed, with 1,708 upregulated genes and 756 downregulated genes (Figure 5E). As expected, key lipogenic transcriptional factor ChREBP and its target genes *Fasn*, *Acc1*, and *Acly* were significantly upregulated (Figure 5F). But, unexpectedly, GSEA revealed that the muscle development pathway was at the top of the significantly upregulated pathways, whereas genes responsible for the mitochondrial respiratory chain were significantly blunted (Figures 5G–I). In support of the GSEA result, quantitative real-time (qRT)-PCR analysis revealed that the expression levels of myogenic signature genes *Myod*, *Myog*, *Mef2c*, *Myh1, Myh4*, and *Myh7* and pro-myogenic genes *Igf2* and *Igf2bp3* were significantly upregulated (Figure 5J), whereas the common adipogenic genes *Pparg*, *Cebpa*, *Adipoq*, and *Fabp4* were downregulated (Figure 5K), interestingly leaving the BAT marker genes *Ucp1*, *Cidea*, *Prdm16*, and *Cebpb* not affected (Figure 5L). Because it has already known that the myogenic pathway is usually inhibited during BAT development and abnormal activation of this pathway leads to BAT dysfunction and metabolic disorders (18, 20, 21, 27), thus our findings suggest that the induction of myogenic signature in fetal BAT partially results in the impairment of the BAT development and function in HF offspring.

### Brown Adipose Tissue Myogenic Programming Can Be Acutely Induced by Fructose Administration

**FIGURE 6:**
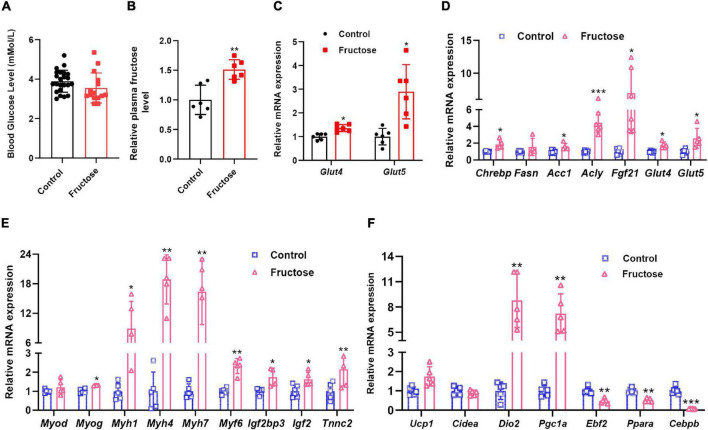
Brown adipose tissue myogenic programming can be acutely induced by fructose administration. **(A)** The blood glucose level in fetuses at E18.5 (*n* = 15–23). **(B)** The relative level of plasma fructose in fetuses at E18.5 (*n* = 6). **(C)** qRT-PCR analysis of *Glut4* and *Glut5* expression in BAT of fetuses at E18.5 (*n* = 6). qRT-PCR analysis of lipogenic genes **(D)**, myogenic genes **(E)**, and thermogenic genes **(F)** in BAT of mice gavaged with fructose or water (*n* = 4–6). *p < 0.05, **p < 0.01, ***p < 0.001.

Next, to further reveal how maternal high-fructose feeding activates fetal BAT myogenic gene program, we first measured the circulating fructose and glucose levels in fetal blood at E18.5. Interestingly, the glucose level was comparable between the two groups (Figure 6A); however, fetal HF offspring showed about 1.5-folds higher plasma fructose levels than the controls (Figure 6B). Moreover, the expression of the fructose transporter gene *Glut5* in BAT was also strikingly upregulated in fetal HF offspring (Figure 6C). These data suggest that maternal-derived fructose may directly play a regulatory role in fetal BAT development. Then, to test whether blood fructose elevation is sufficient to induce myogenic signature in BAT, we fasted male mice for 24 h and then acutely fed mice with fructose or water through gavage. About 6 h later, mice BAT were isolated and analyzed by qRT-PCR. As expected, the acute high-fructose feeding significantly upregulated the levels of *Chrebp* and its target genes (Figure 6D). Strikingly, it also activated the expression of myogenic genes, such as *Myh1, Myh4*, and *Myh7* (Figure 6E). Of note, interestingly, the thermogenic gene was differentially regulated by the acute high-fructose challenge. Specifically, mRNA levels of *Dio2* and *Pgc1a* were dramatically elevated but *Ebf2*, *Ppara*, and *Cebpb* were significantly downregulated (Figure 6F). Overall, these data indicate that dietary fructose could activate BAT myogenic gene program. Taken together, these data support that maternal high-fructose feeding induces fetal BAT myogenic signature probably through a fructose-to-BAT mechanism.

## Discussion

**FIGURE 7:**
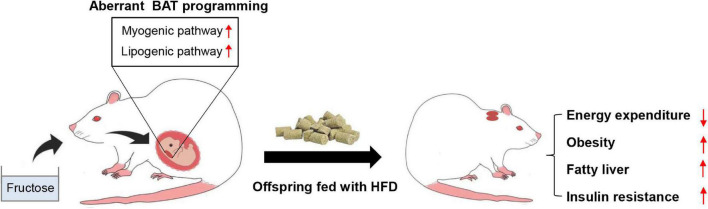
A model depicting the effects of maternal high-fructose feeding on offspring BAT development and metabolic health. Maternal high-fructose feeding during gestation reprograms brown fat lineage specification, especially exhibiting activated myogenic pathway and lipogenic pathway, which probably results in the impairment of HFD-induced energy expenditure and the predisposition of HFD-induced obesity, fatty liver, and insulin resistance in adult offspring. *p < 0.05, **p < 0.01, ***p < 0.001.

In this study, we explored the effects and potential mechanisms underlying maternal high-fructose intake on offspring metabolic health. We found maternal high-fructose feeding predisposed adult offspring to HFD-induced obesity, fatty liver, and insulin resistance. Mechanistically, maternal-derived fructose hampered the fetal BAT development partially *via* inducing a myogenic signature in fetal BAT and resultantly impaired HFD-induced thermogenesis in adult mice (Figure 7). Thus, this study provides evidence for a pregnant woman to control their daily fructose intake. Also, this study reveals a previously uncovered myogenic pathway that probably mediates the detrimental effects of excess fructose consumption on metabolism.

Maternal nutrition plays a pivotal role in determining descendant metabolic health (3); therefore, it is important to clarify the effects of primary nutrients in our daily diet on the health of the offspring, to provide dietary guidance for the pregnant women to protect the health of offspring. Fructose is widely used as a food and drink sweetener, and excess consumption of fructose is considered as a major contributor to the global obesity epidemic (6). However, whether maternal high-fructose intake is also responsible for obesity prevalence remains largely unknown. In this study, we found that offspring delivered from dams fed with high-fructose water were prone to HFD-induced obesity, fatty liver, and insulin resistance (Figure 2). To our knowledge, this is the first report comprehensively describing the effects of maternal high-fructose feeding on adult offspring metabolic health. Interestingly, we noticed that the adverse metabolic consequences caused by maternal high-fructose feeding to adult offspring are similar to that caused by maternal HFD feeding (28, 29), thus our study highlights the importance of the diet intake control of fructose besides fat, especially during the period of pregnancy.

Then, we explored the underlying mechanism from the angle of energy metabolism and found HF offspring showed impaired energy expenditure only on a HFD but not on a CD (Figure 3 and Supplementary Figure 2). This finding not only explains why offspring with a CD do not develop obesity but also indicates that the heat-production organ, namely, BAT, is probably involved in this adverse process. Indeed, histological and molecular examination confirmed that the function of BAT is disrupted in HFD-fed HF offspring (Figures 3, 4). Although we are unable to exclude the contributions of other organs, BAT dysfunction is definitely a major player in the development of obesity and metabolic disorders in HF offspring.

The fetal BAT development has a profound effect on adult BAT function and global energy homeostasis in later life (30, 31). Studies showed that maternal nutrition influences the metabolic health of the adult offspring partially by affecting fetal BAT development. For instance, maternal HFD results in offspring metabolic disorders *via* inhibiting fetal BAT development (28, 32), whereas maternal resveratrol or n-3 PUAF supplementation ameliorates HFD-induced obesity through enhancing offspring BAT development (33, 34). However, to date, whether maternal high-fructose feeding also adversely affects fetal BAT development is still unknown. To this end, we closely examined fetal BAT morphology and gene expression profiles (Figure 5). As expected, maternal high-fructose feeding increased fetal BAT lipid deposition and elevated expression of genes in the *de novo* lipogenesis pathway (Figures 5C,D,F). That is probably because plasma fructose level in fetuses is significantly increased after dams fed with a high-fructose drink (Figure 6B), and it is already known that fructose-derived intermediate metabolites can activate the transcriptional activity of ChREBP, a major lipogenic transcription factor in adipose tissue (35). Of note, it was reported recently that overexpression of *Chrebpb*, a constitutively active isoform of ChREBP (36), exclusively in BAT increased the lipid droplet size and impaired BAT thermogenic function (26). Therefore, activating the ChREBP-mediated pathway in fetal BAT may also partially contribute to the adverse metabolic phenotypes observed in HF offspring. However, unexpectedly, the GSEA result clearly displayed that the most predominantly upregulated pathway in fetal BAT was muscle tissue development but not lipogenesis (Figure 5G). Because the myogenic pathway is usually inhibited during BAT determination (17), and alleviating this inhibition always leads to BAT developmental delay and metabolic disorders in later life (18, 20, 21, 27), thus fructose-induced activation of myogenic programming in fetal BAT could be a leading contributor to the BAT dysfunction and later metabolic disorders.

Currently, we do not know exactly how maternal-derived fructose activates fetal BAT myogenic programming, however, based on the finding that fructose solution gavage acutely activates BAT lipogenic and myogenic pathways (Figures 6D,E), one possibility is that circulating fructose can directly act on the BAT and regulate target genes expression. Of note, fructose can be rapidly catalyzed by Ketohexokinase (KHK) to generate fructose-1-phosphate (F-1-P) once entering cells through transporter GLUT5 (37), thus it could be the intermediate metabolites of fructose but not fructose itself that exerts the regulatory function. Therefore, as the major organs for fructose metabolism, the liver and small intestine may be also involved in the regulation of fetal BAT myogenic programming. To date, although there are no reports regarding fructose intermediate metabolites in regulating myogenesis, we noticed that one recent study showed that myoblast cellular level of fructose-1,6-bisphosphate (F-1-6-P), a glucose intermediate metabolite, maintains the protein stability of MyoD (38), a key myogenic factor. Since F-1-P has a similar structure with F-1-6-P, it is possible that F-1-P may also regulate BAT MyoD protein stability in the same way with F-1-6-P, but more studies are required to test this possibility in the future.

## Conclusion

In summary, we have demonstrated that maternal high-fructose exposure induces myogenic signature in fetal BAT and impairs fetal BAT development and HFD-induced energy expenditure, which predisposes adult offspring to obesogenic diet-induced obesity and metabolic disorders. This study highlights the importance of limiting the intake of fructose-enriched diets in pregnancy to protect offspring metabolic health in later life.

## Data Availability Statement

The datasets presented in this study can be found in online repositories. The names of the repository/repositories and accession number(s) can be found below: https://www.ncbi.nlm.nih.gov/, GSE193031.

## Ethics Statement

The animal study was reviewed and approved by the Animal Ethics Committee of Guangxi University.

## Author Contributions

ZS conceived, designed, and supervised the study. PW and TW performed most of the experiments and data analyses. ZS and PW wrote the manuscript. QF, QL, and YaL participated in the experiments. TH, YiL, and LZ provided scientific advice and discussion. All authors contributed to the article and approved the submitted version.

## Conflict of Interest

The authors declare that the research was conducted in the absence of any commercial or financial relationships that could be construed as a potential conflict of interest.

## Publisher’s Note

All claims expressed in this article are solely those of the authors and do not necessarily represent those of their affiliated organizations, or those of the publisher, the editors and the reviewers. Any product that may be evaluated in this article, or claim that may be made by its manufacturer, is not guaranteed or endorsed by the publisher.

## Footnotes

1
http://www.ncbi.nlm.nih.gov/projects/geo/

## Supplementary Material

Supplementary Figure 1 The effects of maternal high-fructose intake during pregnancy on adult offspring fed with a chow diet (CD). All data were from the CD-fed control offspring and HF offspring. **(A)** Bodyweight curve of the offspring. **(B,C)** Relative body composition of the offspring. **(D)** Tissue weights of the offspring. **(E)** H&E staining of paraffin sections of inguinal white fat (iWAT), epididymal white fat (eWAT), and liver. Scale bar, 100 μm. **(F)** Quantification of average adipocyte area from images depicted in **(E)**. **(G)** Quantification of triglyceride (TG) content in liver (*n* = 8). **(H,I)** Plasma concentrations of TG and total cholesterol (TC) (*n* = 8). **(J)** Blood glucose concentrations during glucose tolerance test (*n* = 6). **(K)** Quantification of the area under the curve of **(J)**. **(L)** Blood glucose concentrations during insulin tolerance test (*n* = 6). **(M)** Quantification of the area under the curve of **(L)**. **p* < 0.05, ***p* < 0.01.

Supplementary Figure 2 Maternal high-fructose intake during pregnancy does not affect energy expenditure in adult offspring with a chow diet. All data were from the 17-week-old CD-fed control offspring and HF offspring. **(A)** Daily food intake of the mice (*n* = 5). Indirect calorimetry analysis of day and night oxygen consumption (VO_2_, **B,C**), carbon dioxide production (VCO_2_, **D,E**), heat production **(F,G)**, and physical activity **(H,I)** of the mice (*n* = 5).
