# Supplementary figures and images for: Maternal High-Fructose Intake Activates Myogenic Program in Fetal Brown Fat and Predisposes Offspring to Diet-Induced Metabolic Dysfunctions in Adulthood
Source: Front Nutr. 2022 Apr 11;9:848983. doi: 10.3389/fnut.2022.848983 (PMC9036479; doi:10.3389/fnut.2022.848983)

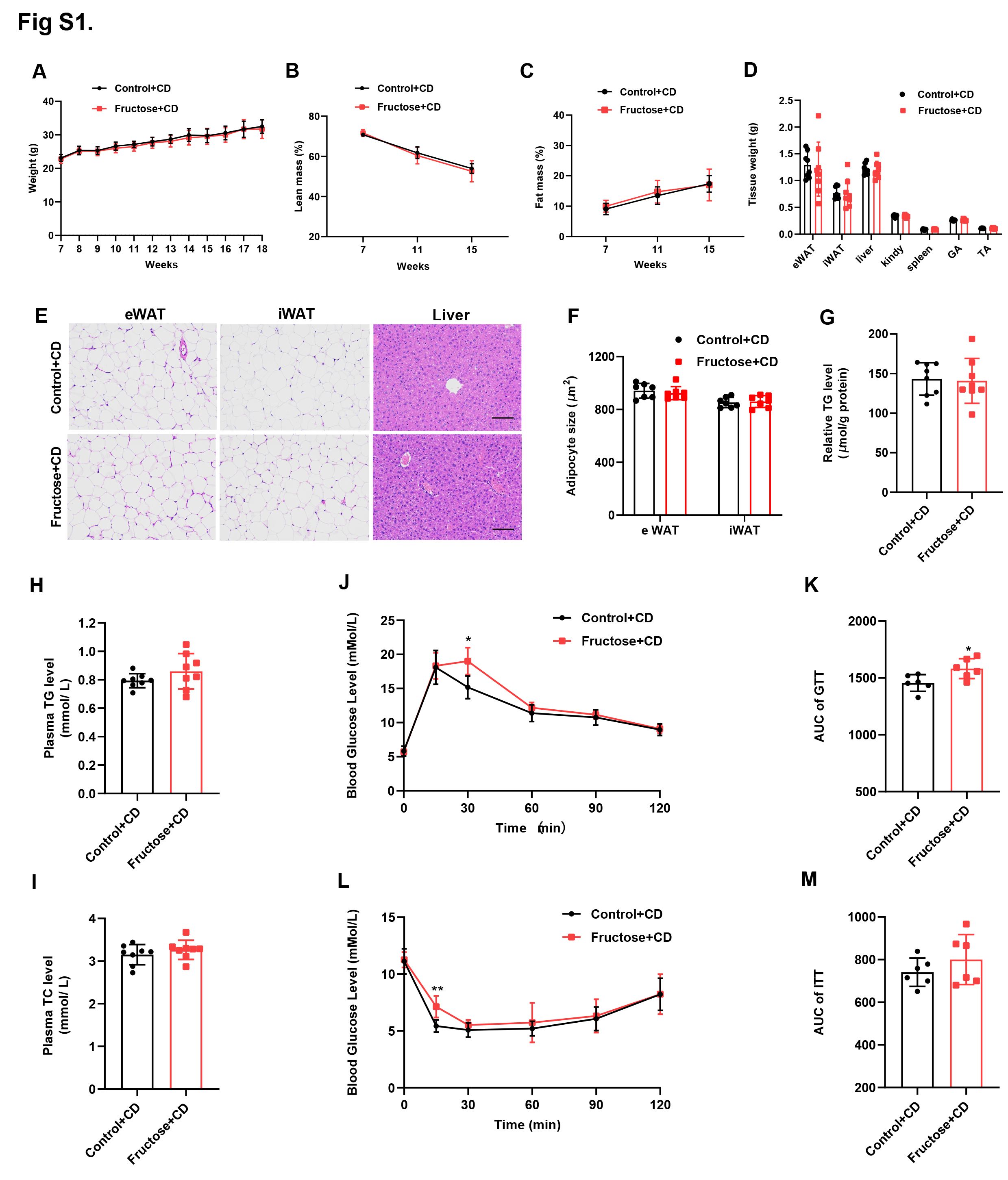

Supplement: Supplementary Figure 1 — The effects of maternal high-fructose intake during pregnancy on adult offspring fed with a chow diet (CD). All data were from the CD-fed control offspring and HF offspring. (A) Bodyweight curve of the offspring. (B,C) Relative body composition of the offspring. (D) Tissue weights of the offspring. (E) H&E staining of paraffin sections of inguinal white fat (iWAT), epididymal white fat (eWAT), and liver. Scale bar, 100 μm. (F) Quantification of average adipocyte area from images depicted in (E). (G) Quantification of triglyceride (TG) content in liver (n = 8). (H,I) Plasma concentrations of TG and total cholesterol (TC) (n = 8). (J) Blood glucose concentrations during glucose tolerance test (n = 6). (K) Quantification of the area under the curve of (J). (L) Blood glucose concentrations during insulin tolerance test (n = 6). (M) Quantification of the area under the curve of (L). *p < 0.05, **p < 0.01. [file Image_1.TIF]

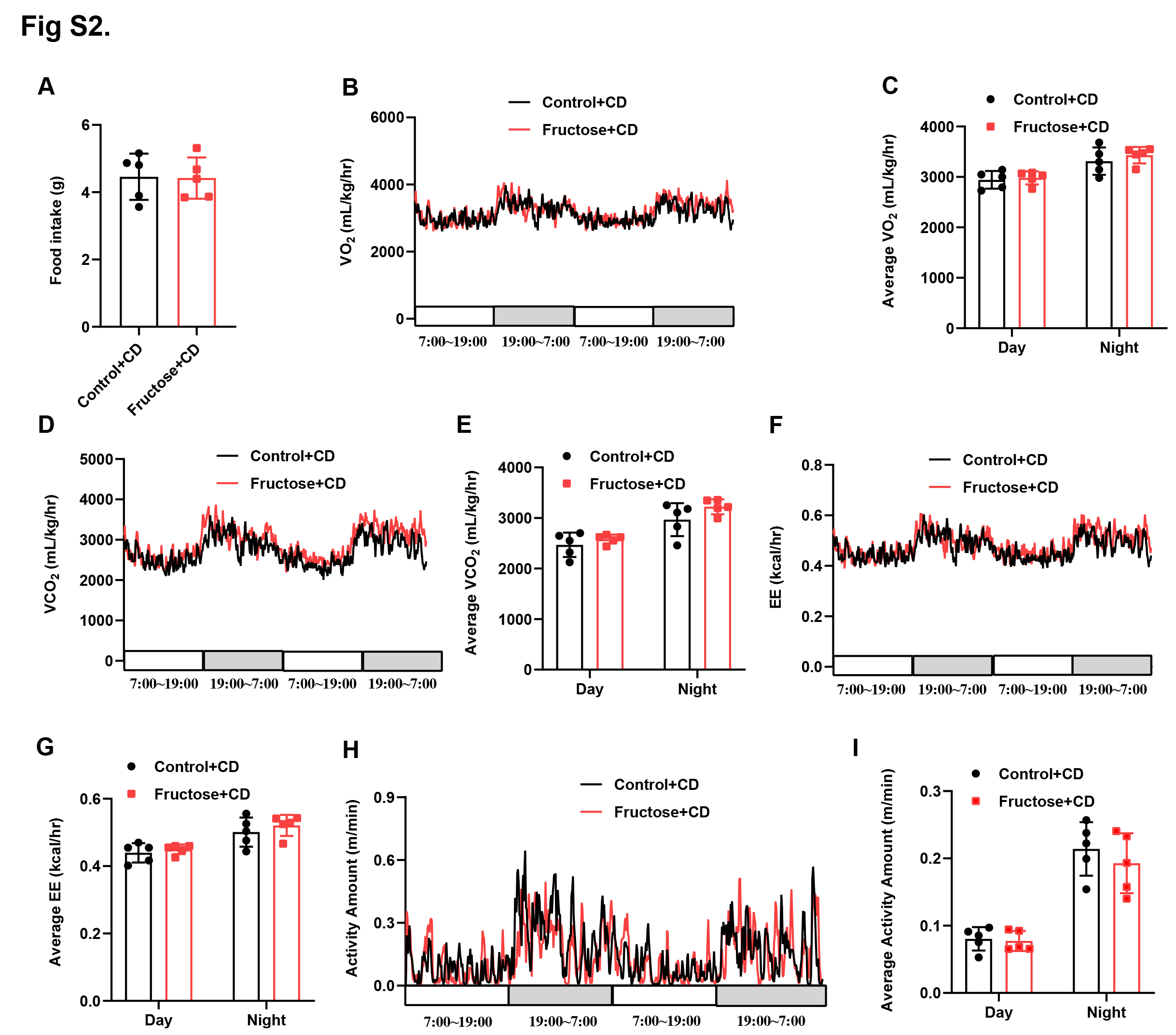

Supplement: Supplementary Figure 2 — Maternal high-fructose intake during pregnancy does not affect energy expenditure in adult offspring with a chow diet. All data were from the 17-week-old CD-fed control offspring and HF offspring. (A) Daily food intake of the mice (n = 5). Indirect calorimetry analysis of day and night oxygen consumption (VO2, B,C), carbon dioxide production (VCO2, D,E), heat production (F,G), and physical activity (H,I) of the mice (n = 5). [file Image_2.TIF]
