# Supplementary material for: Maternal High-Fructose Intake Activates Myogenic Program in Fetal Brown Fat and Predisposes Offspring to Diet-Induced Metabolic Dysfunctions in Adulthood
Source: Front Nutr. 2022 Apr 11;9:848983. doi: 10.3389/fnut.2022.848983 (PMC9036479; doi:10.3389/fnut.2022.848983)
Supplement: Supplementary file 3 [file Table_1.docx]

Supplementary Table 1. The sequence of qPCR primers used in this study.

| **The sequence of qPCR primers** | | | |
| --- | --- | --- | --- |
| **Gene** | **Speices** | **Forward Primer (5'-3')** | **Reverse Primer (5'-3')** |
| *Acc1* | Mouse | CCGAGAAAGCAGGGGATCTG | TACCCGACGCATGGTTTTCA |
| *Ac**ly* | Mouse | GCTGTGCAGGGCATGCTGGA | CCATGGCAGCCACTGAGGGC |
| *Adipoq* | Mouse | GCCGCTTATGTGTATCGCTCAG | TTGCCAGTGCTGCCGTCATA |
| *Cebpa* | Mouse | TGGACAAGAACAGCAACGAG | TCACTGGTCAACTCCAGCAC |
| *Cebpb* | Mouse | GACTGACGCAACACACGTGTAA | ATCAACAACCCCGCAGGAA |
| *Chrebp* | Mouse | GCTCAACGCTGCCATCAACTTG | GGTCGGATGAGGATGCTGAACA |
| *Chrebpa* | Mouse | ATCCGACACTCACCCACCTCTT | GCTCTCCAGATGGCGTTGTTCA |
| *Chrebpb* | Mouse | TCTGCAGATCGCGTGGAG | CTTGTCCCGGCATAGCAAC |
| *Cidea* | Mouse | ACCTTAAGGGACAACACGCAT | TTTGGTTGCTTGCAGACTGG |
| *Cox5b* | Mouse | GCTGCATCTGTGAAGAGGACAAC | CAGCTTGTAATGGGTTCCACAGT |
| *Cox8b* | Mouse | GAACCATGAAGCCAACGACT | GCGAAGTTCACAGTGGTTCC |
| *Cpt1b* | Mouse | TGTCTACCTCCGAAGCAGGA | TGAACGGCATTGCCTAGACG |
| *Cyt-c* | Mouse | GCAAGCATAAGACTGGACCAAA | TTGTTGGCATCTGTGTAAGAGAATC |
| *Dio2* | Mouse | CAGTGTGGTGCACGTCTCCAATC | TGAACCAAAGTTGACCACCAG |
| *Ebf2* | Mouse | ATCCGCAACACAAGCAGCATCT | CCGTGGGTGAGCCATTCAGAAA |
| *Fabp4* | Mouse | AAGAAGTGGGAGTGGGCTTTG | CTCTTCACCTTCCTGTCGTCTG |
| *Fasn* | Mouse | CACTGCATTGACGGCCGGGT | GGACAAGCCCAGGCTGCGAG |
| *Fgf21* | Mouse | TCCAGTTTGGGGGTCAAGTC | ACCACTGTTCCATCCTCCCT |
| *Glut4* | Mouse | GTCCTCCTGCTTGGCTTCTTCA | ACTGGGTTTCACCTCCTGCTCT |
| *Glut5* | Mouse | AGAGCAACGATGGAGGAAAA | CCAGAGCAAGGACCAATGTC |
| *Igf2* | Mouse | AGGATCAACCGTGGCATTGT | TCTGACTTGACGGACTTGGC |
| *Igf2bp3* | Mouse | GCTGCTGCTGCTTCATATCCAC | CCTGCTTGCCAATAATAGCTCCA |
| *Mef2c* | Mouse | ATCCCGATGCAGACGATTCAG | AACAGCACACAATCTTTGCCT |
| *Myf6* | Mouse | GCTAAGGAAGGAGGAGCAAA | GAAGAAAGGCGCTGAAGACT |
| *Myh1* | Mouse | GGACCCACGGTCGAAGTTG | CCCGAAAACGGCCATCT |
| *Myh4* | Mouse | CAATCAGGAACCTTCGGAACAC | GTCCTGGCCTCTGAGAGCAT |
| *Myh7* | Mouse | ATGAGCTGGAGGCTGAGCA | TGCAGCCGCAGTAGGTTCTT |
| *Myod* | Mouse | GGCTCTCTCTGCTCCTTTGA | GTAGGGAAGTGTGCGTGCTC |
| *Myog* | Mouse | AGGAAGTCTGTGTCGGTGGA | AGGCGCTCAATGTACTGGAT |
| *Pgc1a* | Mouse | AGCCGTGACCACTGACAACGAG | GCTGCATGGTTCTGAGTGCTAAG |
| *Ppara* | Mouse | CCGGGAGGCGTTTCCTGAG | CAGGCCACAGAGCGCTAA |
| *Pparg* | Mouse | CCAAGAATACCAAAGTGCGATCA | CCCACAGACTCGGCACTCAAT |
| *Prdm16* | Mouse | AGCTGAGGAAGCATTTGAAGT | GAGAGGGACAGCATCATTGC |
| *Psat1* | Mouse | TACCGCCTTGTCAAGAAACC | AGTGGAGCGCCAGAATAGAA |
| *Resistin* | Mouse | CTGTCCAGTCTATCCTTGCACAC | CAGAAGGCACAGCAGTCTTGA |
| *Serpin3ak* | Mouse | GGCTGAAGGCAAAGTCAGTGT | TGGAATCTGTCCTGCTGTCCT |
| *Tnnc2* | Mouse | CGGCTCCATCGACTTTGAAG | AGCAGCTCATCGATCTCCTC |
| *Ucp1* | Mouse | TACCCAAGCGTACCAAGCTG | ACCCGAGTCGCAGAAAAGAA |
